# The Relationship between Lutein and Zeaxanthin Status and Body Fat

**DOI:** 10.3390/nu5030750

**Published:** 2013-03-08

**Authors:** Emily R. Bovier, Richard D. Lewis, Billy R. Hammond

**Affiliations:** 1 Vision Sciences Laboratory, University of Georgia, Athens, GA 30602, USA; E-Mail: erbovier@uga.edu; 2 Foods and Nutrition, University of Georgia, Athens, GA 30602, USA; E-Mail: rlewis@fcs.uga.edu

**Keywords:** lutein, zeaxanthin, body fat, adiposity

## Abstract

The objective of this project was to investigate the relationships between total and regional distribution of body fat and tissue lutein (L) and zeaxanthin (Z) status. Healthy men and women (*N* = 100; average age: 22.5 year, average BMI: 23.4 kg/m^2^) were evaluated. Total body and regional fat mass were assessed by dual-energy X-ray absorptiometry (Hologic Delphi A). Serum LZ was measured using reverse phase high-performance liquid chromatography, and retinal LZ (referred to as macular pigment optical density; MPOD) was measured using heterochromatic flicker photometry. Body fat percentage (total and regional) was inversely related to MPOD (*p* < 0.01) but no significant relationship was found for serum LZ. Higher body fat percentage, even within relatively healthy limits, is associated with lower tissue LZ status. The results indicate that adiposity may affect the nutritional state of the retina. Such links may be one of the reasons that obesity promotes age-related degenerative conditions of the retina.

## 1. Introduction

Lutein and zeaxanthin (LZ), two lipophilic pigments obtained exclusively from the diet, are likely to have a constellation of health effects throughout the body. Higher tissue concentrations of LZ have been associated with a reduced risk of acquired diseases, including cardiovascular disease, various cancers, and age-related eye diseases [1]. LZ status can be quantified by measuring concentrations within the serum and, unlike most nutrients, by noninvasive measurements within tissue. For example, a variety of physical and psychophysical methods are available to directly measure LZ concentrations within the central macular region of the human retina [2]. In the macula, the area that contains the highest carotenoid concentrations within the body, LZ (and a third isomeric intermediary, meso-zeaxanthin) are termed macular pigment (MP). Preliminary evidence has shown that MP correlates with amounts of LZ in adipose tissue, which is a major body store for LZ [3,4,5]. 

Although dietary intake is the primary driver of tissue LZ levels, the relation between diet and the ultimate deposition of these pigments within a target tissue is moderated by a number of factors. Hammond *et al.* [6] for instance, found significantly lower MP for women compared to men, despite equivalent plasma concentrations and dietary intakes of LZ. The authors concluded that this relative paucity in women could help explain the higher incidence of age-related eye diseases directly associated with serum LZ and MP, such as age-related macular degeneration and cataracts. Although males and females obviously differ across many biological dimensions, one possible reason for the observed sex differences in MP [5,6,7] might be the fact that women generally have higher body fat percentages than men. Higher levels of body fat have been shown to be related to lower levels of circulating carotenoids (The reverse is likely also true. Individuals with very low body fat (like anorexics who would then have lower capacity for adipose storage of carotenoids) often have higher circulating carotenoid levels [8]), making these pigments less available to retinal tissue [9]. Consistent with this interpretation, higher body fat levels, especially when approaching obesity, have also been linked to lower levels of MP. Hammond *et al.* [10], for instance, found that both men and women with body fat greater than 27% had 16% lower MP density compared to subjects with lower body fat. One question that was not addressed by Hammond *et al.* is whether the distribution of body fat (also known to differ between men and women [5]) influences serum LZ and/or the ultimate deposition of these pigments within the retina. Chung *et al.* [11] have shown that LZ concentrations in adipose tissue differ according to body site (e.g., levels tend to be higher in the abdomen than in the buttocks). 

The primary goal of the present study was to assess the relation between LZ status (measured in serum and retina) to total and regional fat distribution (focusing on the trunk).

## 2. Methods

### 2.1. Subjects

Male (*N* = 39) and female (*N* = 61) subjects (average age of 22.5 years; average BMI of 23.4 kg/m^2^) were recruited from The University of Georgia and the surrounding Athens area. Subjects were screened for ocular health (e.g., no history of corneal disease, age-related macular degeneration, *etc.*) and corrected visual acuity better than 20/60. All subjects completed informed consent and two measures of MP (an average over the two visits was used in all subsequent analyses) at the Vision Sciences Laboratory. Within two to four weeks, subjects completed assessments of body composition at the UGA Bone Clinic. Serum LZ was determined for 65 subjects. Experimental procedures were approved by the Institutional Review Board of The University of Georgia. 

### 2.2. Assessment of LZ Status: Macular Pigment Optical Density & Serum LZ

Heterochromatic flicker photometry (HFP) was used to measure the optical density of the macular pigments (*i.e.*, macular pigment optical density; MPOD) [12]. This method has been extensively validated [2,13] and is fully described by Wooten *et al.* [12]. Briefly, we used the standardized CAREDs protocol [14], which involves measuring sensitivity to stimuli presented in free-view. A target stimulus alternates in square-wave between a “blue” light maximally absorbed by MP (460 nm) and a “green” light not absorbed by MP (540 nm). Given the differential absorbance of “blue” light compared to “green” light, the discordance in the amount of energy that reaches the photoreceptors is perceived as a flicker. Subjects’ thresholds were obtained by having the subjects minimize or eliminate the perception of flicker, a condition known as sensation luminance [15], by adjusting the radiance of the 460 nm light (while the radiance of the 540 nm light was held constant) until the energy of the two lights were perceptually the same. Since flicker sensitivity also differs across subjects, the flicker alternation rates were optimized for each subject using the algorithm described in Stringham *et al.* [13] MPOD was measured at 15′, 30′, 60′, and 105′ retinal eccentricity in the temporal hemi-retina with a macular densitometer, manufactured by Macular Metrics (Rehoboth, MA, USA). 

Assessment of serum LZ required collection of blood into 10 mL lithium heparin coated vacutainers (BD) by a licensed phlebotomist. Plasma was separated by centrifugation at 1500× *g* for 20 min at 4 °C and then distributed into light protected Eppendorf vials tubes for storage at −80 °C. The analysis of the blood was done by the analytical laboratories of DSM Nutritional Products Ltd., Kaiseraugst, Switzerland. Serum LZ were quantified with a normal-phase HPLC system after extraction with a *n*-hexane/chlorophorm 20% (v/v) mixture. 

### 2.3. Assessment of Body Composition

Fat mass (g), percentage body fat, and fat-free soft tissue mass (FFST; g) were assessed using dual-energy X-ray absorptiometry (DXA; Delphi A; S/N 70467; Hologic Inc., Bedford, MA, USA). All scans were analyzed by the same technician with the use of Hologic software, version 11.2. Quality assurance for body composition variables was performed by calibration against a 3-step soft tissue wedge (model TBAR; SN 2275) composed of variable thicknesses of aluminum and lucite, calibrated against stearic acid (100% fat) and water (8.6% fat). With respect to test-retest reliability, single intraclass correlation coefficients (ICCs) were calculated from ten females (aged 18 to 30 years) scanned twice within seven days (*R* ≥ 0.87).

### 2.4. Statistical Analyses

Pearson-product moment correlations were conducted to determine associations between LZ status and fat mass, percentage body fat, and FFST. Comparisons between male and female subjects were made with independent samples *t*-tests. Statistical significance was set at *p* < 0.05.

## 3. Results

The descriptive data for body composition and LZ status stratified by sex are listed in Table 1. The average MPOD at each eccentricity across the retina for the sample was 0.53 at 15′ eccentricity, 0.43 at 30′, 0.29 at 60′, and 0.13 at 105′ eccentricity. Mean serum LZ levels (for the truncated sample, *n* = 65) were 0.26 μmol/L. Women had higher serum LZ than men (0.28 μmol/L compared to 0.21 μmol/L; *t* (63) = 2.52, *p* = 0.01), however, despite the absence of differences in MPOD (see Table 1). 

The average body fat percentage was approximately 26%. Women had significantly higher body fat percentage than men (30% compared to 19%; *t* (98) = 10.52, *p* < 0.01). This discrepancy in body fat percentage between men and women was consistent for each region of the body (see Table 1). However, with respect to the amount of fat in the trunk region relative to the rest of the body, men accumulated a higher percentage of total body fat than women (approximately 48% compared to 43%; *t* (98) = 3.53, *p* = 0.001).

**Table 1:** Means and standard deviations for body composition variables and LZ status.

	Entire Sample (*N* = 100)	Males (*N* = 39)	Females (*N* = 61)
**Body Fat Percentage**			
Total Body	25.79 ± 7.60	18.90 ± 4.89	30.19 ± 5.45
Leg	30.05 ± 8.98	20.72 ± 4.86	36.01 ± 5.03
Trunk	23.12 ± 7.95	17.81 ± 6.15	26.51 ± 7.09
Arm	26.15 ± 9.80	16.44 ± 4.47	32.35 ± 6.75
Trunk Fat (Relative) ^a^	44.86 ± 6.35	47.52 ± 6.41	43.17 ± 5.76
**Body Mass (g)**			
Total Body	65,346 ± 13,345	72,423 ± 12,516	60,790 ± 11,853
Fat-Free Soft Tissue	48,796 ± 10,413	58,980 ± 7291	42,284 ± 5891
Total Body Fat	17,062 ± 7090	13,847 ± 6062	19,132 ± 6972
**LZ Status**			
MPOD 15′	0.53 ± 0.21	0.55 ± 0.20	0.52 ± 0.22
MPOD 30′	0.43 ± 0.18	0.45 ± 0.17	0.42 ± 0.19
MPOD 60′	0.29 ± 0.14	0.29 ± 0.13	0.29 ± 0.14
MPOD 105′	0.13 ± 0.09	0.14 ± 0.09	0.12 ± 0.09
Serum LZ^ b^	0.26 ± 0.12	0.21 ± 0.07	0.28 ± 0.14

^a ^Refers to the percentage of total body fat in the trunk region; ^b ^*N* = 65 (39 Females, 26 Males).

**Figure 1:**
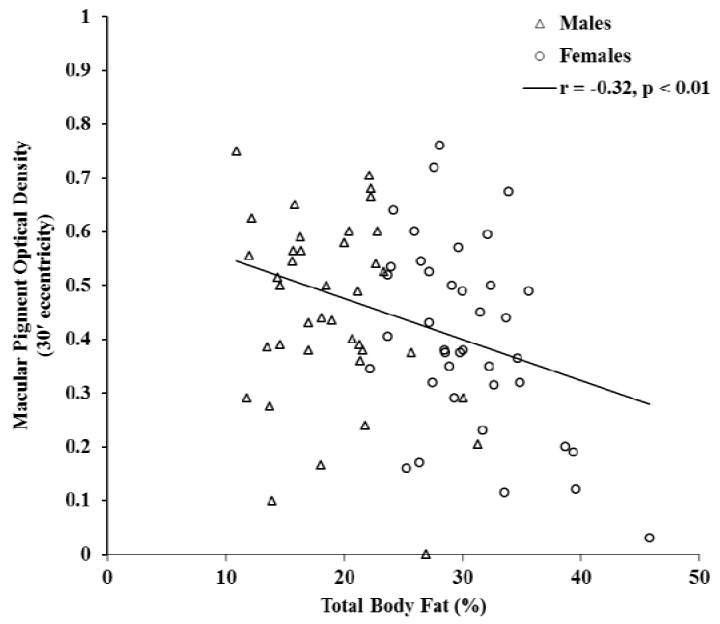
The relationship between MPOD at 30'eccentricity and total body fat percentage.

**Figure 2:**
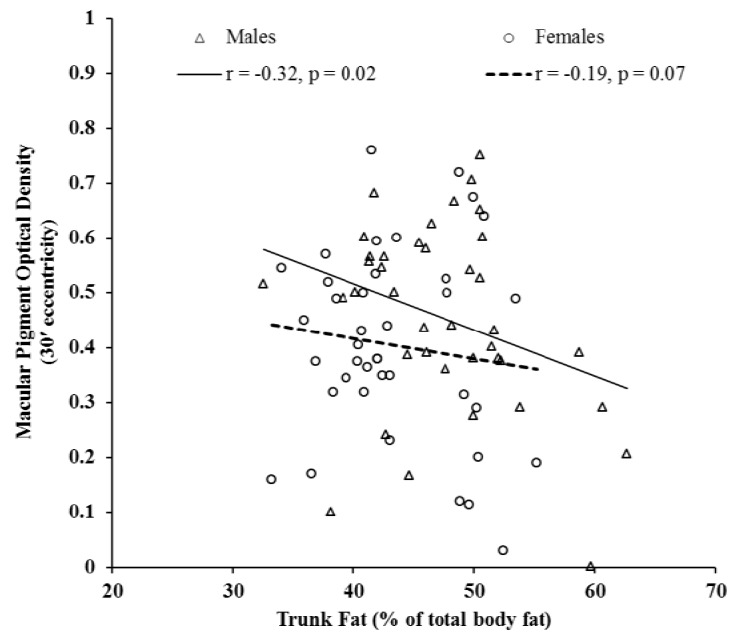
The relationship between MPOD at 30' eccentricity and the percentage of total body fat that accumulated in the trunk region for male (*N* = 39) and female (*N* = 61) subjects.

Figure 1 illustrates the significant inverse relationship between total body fat percentage and MPOD at 30′ eccentricity (*r* = −0.32, *p* < 0.01). This relationship was consistent for each region of the body. Furthermore, individuals with higher body fat percentage had lower MPOD at each retinal eccentricity (see Table 2). The relationship between MPOD and the amount of fat in the trunk region relative to the rest of the body was statistically significant (*r* = −0.20, *p* = 0.05). However, when analyses were performed for men and women separately, statistical significance remained for men (*r* = −0.32, *p* = 0.02) but not for women (*r* = −0.19, *p* = 0.07), as shown in Figure 2. Consistent with its relation to body fat percentage, MPOD at each retinal eccentricity was related to fat mass (*p* < 0.05). Serum LZ was not significantly related to body fat (see Table 2). MPOD and serum LZ were not significantly related to fat-free soft tissue mass (data not reported).

**Table 2:** Pearson-product moment correlation coefficients for associations between body fat percentage and LZ status.

	Body Fat Percentage				
	Total	Leg	Trunk	Arm	Trunk (Relative)^ a^
MPOD 15′	−0.26 *	−0.18	−0.28 **	−0.25 *	−0.10
MPOD 30′	−0.32 **	−0.22 *	−0.37 **	−0.30 **	−0.20 *
MPOD 60′	−0.24 *	−0.14	−0.31 **	−0.21 *	−0.23 *
MPOD 105′	−0.29 **	−0.20 *	−0.32 **	−0.28 **	−0.16
Serum LZ	0.16	0.15	0.16	0.11	−0.02

** *p* < 0.01; * *p* < 0.05; ^a ^Refers to the percentage of total body fat in the trunk region.

## 4. Discussion

Consistent with past studies [7,10,16], the primary result of the present study was an inverse relation between MP and body fat across most sites measured. This effect, although statistically significant, and also like past studies of this relation, was only moderate. This may be attributed to the use of young healthy subjects with very limited variability in diet and adiposity: to wit, the group least likely to show effects. Often nutritional relations are driven by the extremes (e.g., those deficient in intake, those showing loss, *etc.*). For example, an inverse relationship between MP and body fat was driven by obese subjects in a study by Hammond *et al.* [10], since the findings were for subjects with over 27% body fat. Limiting our data collection to subjects within a normal range of adiposity, however, was purposeful. Market studies indicate that 85% of Americans take supplements and that these are most often individuals who are already healthy and tend to be affluent and young [17]. We wanted to assess whether normal variation in body fat was related to tissue LZ status within the average range of most Americans, and to determine differences between men and women. 

An interaction between adipose and retinal tissue in lutein (L) metabolism was originally proposed by Johnson *et al.* [5] based on findings that changes in adipose L concentration were linked to changes in MP. This relationship, however, was specific to sex: that is, significant negative correlations were found between adipose tissue L concentrations and MP for women, but a significant positive relation was found for men. Broekmans *et al.* [7] reported 13% lower MP in females, despite significantly higher serum and adipose tissue L, and positive associations of adipose L with serum L and MP for male subjects. Nolan *et al.* [16] have argued that their data are consistent with a competition between the retina and body fat for LZ, but only for males. In their sample, MP was inversely related to body fat percentage for men, but no effect was found for women. The results of the current study indicate that higher body percentage, for both men and women, is related to lower MP. This relationship was consistent for each region of the body. However, total fat in the trunk region (relative to the rest of the body) was related to MP in men but not in women. Chung *et al.* [11] reported higher LZ in abdominal adipose tissue compared to buttocks and thighs, and a stronger relationship between LZ in serum and adipose tissue in the abdomen compared to other sites. Our data support the idea that the distribution of body fat, specifically in the abdominal region, influences retinal accumulation of LZ, and that this mechanism may account for differences for men and women in the relationship between adipose and LZ tissue status. This has implications for individual differences in the effectiveness of dietary interventions with LZ. 

Dietary supplements containing LZ are being marketed as a way to increase general wellness and protect against conditions linked to oxidative stress. This market trend has been motivated by a large wealth of scientific data showing that a diet deficient in antioxidants is likely to be associated with an elevated risk of degenerative damage. Hammond *et al.* [18] was perhaps the first to note, however, that the same dietary intervention can have significantly different retinal effects across individuals. In that study, some subjects responded vigorously (*i.e.*, tissue LZ increased strongly) to dietary modification with 12 mg of LZ per day (in spinach and corn). Others, however, had no or muted responses. 

The nutritional state of the retina has been consistently linked with adiposity. Obesity has also been consistently linked to higher risk of age-related degenerative eye diseases such as macular degeneration [19,20]. Our data suggest that this link can be explained not only by direct stress effects (such as increased inflammatory or oxidative stress) but also by the reduction of some of the natural tissue defenses we evolved to depend upon, such as optimal nutritional status in the form of sufficient LZ concentrations. 

## References

[1] Mares-Perlman J.A., Millen A.E., Ficek T.L., Hankinson S.E. (2002). The body of evidence to support a protective role for lutein and zeaxanthin in delaying chronic disease. J. Nutr..

[2] Hammond B.R., Wooten B.R., Smollon B. (2005). Assessment of the validity of *in vivo* methods ofmeasuring human macular pigment optical density. Optom. Vis. Sci..

[3] Parker R.S. (1989). Carotenoids in Human Blood and Tissues. J. Nutr..

[4] Kaplan L.A., Lau J.M., Stein E.A. (1990). Carotenoid consumption, concentrations, and relationships in various humans organs. Clin. Physiol. Biochem..

[5] Johnson E.J., Hammond B.R., Yeum K., Qin J., Wang X.D., Castaneda C., Snodderly D.M., Russell R.M. (2000). Relation among serum and tissue concentrations of lutein and zeaxanthin and macular pigment density. Am. J. Clin. Nutr..

[6] Hammond B.R., Curran-Celentano J., Judd S., Fuld K., Krinsky N.I., Wooten B.R., Snodderly D.M. (1996). Sex differences in macular pigment optical density: Relation to plasma carotenoid concentrations and dietary patterns. Vis. Res..

[7] Broekmans W., Berendschot T., Klopping-Ketelaars I., de Vries A.J., Goldbohm R.A., Tijburg L., Kardinaal A.F., van Poppel G. (2002). Macular pigment density in relation to serum and adipose tissue concentrations of lutein and serum concentrations of zeaxanthin. Am. J. Clin. Nutr..

[8] Curran-Celentano J., Erdman J., Nelson R.A., Grater S.J.E. (1985). Alterations in vitamin A, and thyroid hormone status in anorexia nervosa and associated disorders. Am. J. Clin. Nutr..

[9] Gruber M., Chappell R., Millen A., LaRowe T., Moeller S.M., Iannaccone A. (2004). Correlates of serum lutein plus zeaxanthin: Findings from the third national health and nutrition examination survey. J. Nutr..

[10] Hammond B.R., Ciulla T.A., Snodderly D.M. (2002). Macular pigment density is reduced in obese subjects. Invest. Ophthalmol. Vis. Sci..

[11] Chung H., Ferreira A.L.A., Epstein S., Paiva S.A.R., Casteneda-Sceppa C., Johnson E.J. (2009). Site specific concentrations of carotenoids in adipose tissue: Relations with dietary and serum carotenoids concentrations in healthy adults. Am. J. Clin. Nutr..

[12] Wooten B.R., Hammond B.R., Land R.L., Snodderly D.M. (1999). A practical method for measuring macular pigment optical density. Invest. Ophthalmol. Vis. Sci..

[13] Stringham J.M., Hammond B.R., Nolan J.M., Wooten B.R., Mammend A., Smollen W., Snodderly D.M. (2008). The utility of using customized heterochromatic flicker photometry (cHFP) tomeasure macular pigment in patients with age-related macular degeneration. Exp. Eye Res..

[14] Mares J.A., LaRowe D., Snodderly M., Moeller S.M., Gruber M.J., Klein M.L., Wooten B.R., Johnson E.J., Chappell R.J., CAREDS Macular Pigment Study Group and Investigators (2006). Predictors of optical density of lutein and zeaxanthin in retinas of older women in the Carotenoids in Age-Related Eye Disease Study, an ancillary study of the Women’s Health Initiative. Am. J. Clin. Nutr..

[15] Kaiser P.K. (1988). Sensation luminance: A new name to distinguish CIE luminance from luminance dependent on an individual’s spectral sensitivity. Vis. Res..

[16] Nolan J., O’Donovan O., Kavanagh H., Stack J., Harrison M., Muldoon A., Mellerio J., Beatty S. (2004). Macular pigment and percentage of body fat. Invest. Ophthalmol. Vis. Sci..

[17] Dietary Supplement Barometer Survey, The Natural Marketing Institute, 2005. http://www.naturalhealthvillage.com/newsletter/15oct05/264-DSEA_Supplement_Barometer_Survey_Executive%201%20.pdf.

[18] Hammond B.R., Johnson E.J., Russell R.M., Krinsky N.I., Yeum K.J., Edwards R.B., Snodderly D.M. (1997). Dietary modification of human macular pigment density. Invest. Ophthalmol. Vis. Sci..

[19] Johnson E.J. (2005). Obesity, lutein metabolism, and age-related macular degeneration: A web of connections. Nutr. Rev..

[20] Seddon J.M., Cote J., Davis N., Rosner B. (2003). Progression of age-related macular degeneration: Association with body mass index, waist circumference, and waist-hip ratio. Arch. Ophthalmol..

